# Glypican 3 as a Serum Marker for Hepatoblastoma

**DOI:** 10.1038/srep45932

**Published:** 2017-04-05

**Authors:** Shengmei Zhou, Maurice R. G. O’Gorman, Fusheng Yang, Kevin Andresen, Larry Wang

**Affiliations:** 1 Department of Pathology and Laboratory Medicine, Children’s Hospital Los Angeles, Los Angeles, CA, USA; 2 Keck School of Medicine of University of Southern California, Los Angeles, CA, USA.

## Abstract

Hepatoblastoma (HB) is the most common primary liver cancer in children. The conventional serum marker for HB, alpha-fetoprotein (AFP), has its limitations. Novel serum markers need to be explored. Glypican 3 (GPC3) has been reported to be an excellent histological immunomarker for HB. However, the clinical value of serum GPC3 in patients with HB is unknown. A total of 184 serum samples were tested for both GPC3 by ELISA, and AFP by immunometric assay. Of these, 134 were from 32 patients with HB at three treatment stages, 30 from age-matched patients with benign hepatobiliary disorders (BHD) and 20 from age-matched “normal controls”(NC). We found that the GPC3 levels in HB pretreatment group were significantly higher than those in NC group and HB remission group but not statistically different from those in BHD group and HB during treatment group. In contrast, AFP showed significant differences among different groups. The areas under the receiver operating curve (AUROC) value, sensitivity and specificity of GPC3 for HB pretreatment group versus all controls were all significantly lower than those of AFP. Serum GPC3 levels were not associated with prognostic parameters. We concluded that GPC3 is inferior to AFP as a serum marker for HB.

Hepatoblastoma (HB) is the most common primary malignant liver tumor in children worldwide. The incidence of HB in the United States has been increasing^1^. Although complete tumor resection is necessary for long-term disease free survival, less than 50% of cases are resectable at the time of diagnosis^2^, with only 7% of HB patients with pure fetal component being cured by resection alone^1^,^3^. The majority of patients require both resection and systemic chemotherapy. The overall 5-year survival is around 70–90%^4^,^5^. The 3-year survival for patients with progressive or relapsed disease is less than 50% even with intensified chemotherapy^6^,^7^. Side effects of chemotherapy in HB patients are common including hearing loss, cardiomyopathy, nephrotoxicity, bone marrow depression, and second malignancy. Therefore, it is critical to diagnose the disease at an early stage and to identify high-risk patients who would be most likely to benefit from chemotherapy and to spare the low-risk patients from the side effects associated with chemotherapy. The most widely used serum HB marker, alpha-fetoprotein (AFP), has its limitations. First, young infants often have physiologically high levels of AFP^8^. Wu *et al*.^9^ found that the AFP level in normal infants does not decline to the adult level until they are 8 months old. Second, benign liver lesions such as hepatitis and hemangioendothelioma can have elevated AFP levels, partly due to accelerated cellular proliferation and regeneration^10^,^11^. Third, though most HB patients show markedly elevated AFP at the time of diagnosis, unexpectedly low or even normal AFP values were reported in around 5–10% of cases^12^. Fourth, AFP serum levels do not correlate well with the clinical outcome. Therefore, a more sensitive and specific serum marker is clinically needed.

Glypican 3 (GPC3) is an oncofetal protein that is overexpressed in HB^13^ and hepatocellular carcinoma (HCC)^14^. By immunohistochemical staining, we analyzed the expression of five biomarkers including GPC3, beta-catenin, claudin 1, forkhead box protein G1 and delta-like protein in 45 HB cases^15^. We found that GPC3 is a reliable immunomarker for HB with distinctive staining patterns in different epithelial components. Most importantly, it is the only marker among the five to show complete negativity in the surrounding non-neoplastic liver parenchyma. Serum GPC3 is reported to be detectable in 40 to 53% of HCC patients, in approximately one third of patients with normal AFP levels^16^,^17^,^18^, and undetectable in healthy donors^16^,^18^. These findings suggest that serum GPC3 might be an ideal novel tumor marker for HB. To date, no data is available regarding serum GPC3 levels in HB patients. We hypothesized that serum GPC3 is superior to AFP as a diagnostic and monitoring marker for HB.

## Methods

### Patients and serum samples

This study was approved by Children’s Hospital Los Angeles institutional review board (CCI-13-00078) with written informed consent being waived as the research involves no more than minimal risk to subjects. All methods were carried out in accordance with relevant guidelines and regulations. A total of 184 serum samples were collected between June 2013 and July 2016. Of these, 134 were from 32 HB patients at different treatment stages (22 from pretreatment, 70 during treatment and 42 in posttreatment clinical remission), 30 from age-matched patients with benign hepatobiliary disorders (BHD) and 20 from age-matched “normal controls” (NC). “Normal controls” were defined as those with normal liver biochemical function and no evidence of liver disease or germ cell tumors. Each serum sample has a AFP level measured by immunometric assay with VITROS 5600 system (Ortho Clinical diagnostics, Rochester, NY, USA) using standard protocols. The remaining serum samples were aliquoted, coded, and stored at −80 °C until analysis of GPC3. All HB cases were confirmed by histologic examination of tumor tissue including positive immunohistochemical staining for GPC3. Clinicopathological data were collected by retrospective medical record review.

### Measurement of serum GPC3 levels

GPC3 levels were measured with a human GPC3 DuoSet enzyme-linked immunosorbent assay (ELISA) development kit (catalog number: DY2119; R&D Systems, Minneapolis, MN, USA) following the manufacturer’s instructions^19^. Briefly, a 96-well microplate was coated with the monoclonal antibody to GPC3 and incubated overnight, and then washed. After blocking plates for 1 hour, the samples, standards and appropriate controls were added and incubated for 2 hours. After washing away any unbound substances, a detection antibody was added and incubated for 2 hours, followed by the addition of streptavidin-horseradish peroxidase for 20 minutes, color development for about 10 minutes, and then the reaction was stopped with stop solution. Finally, the optical density was determined immediately at 450 nm and referenced to 546 nm on the DS2 spectrophotometer (DYNEX Technologies, Chantilly, VA, USA). All procedures were performed at room temperature and all samples were measured in triplicate. The intra-assay coefficient of variation (CV) and interassay CV of the kit were found to be less than 5% and 10%, respectively (data not shown).

### Statistical analysis

Data were expressed as the median and ranges. Kruskal-Wallis was used to examine the difference among three groups. Wilcoxon rank-sum test with Bonferroni correction was used for multiple pair-wise comparisons. Receiver–operating characteristics (ROC) curves were performed to define the optimal cut-off values, and to assess sensitivity, specificity, and respective areas under the curve (AUCs) with 95% confidence interval (CI). Differences between two independent groups were compared with the Mann-Whitney *U* test. The Spearman’ rank correlation coefficient was used to evaluate the correlation between GPC3 and AFP. The statistical analyses were conducted using Stata 13.1 (StataCorp, college station, Texas) and SPSS, version 17 (International Business Machine (IBM), Armonk, New York). Statistical significance was set at 2-sided 5% level for group comparisons, whereas multiple pair-wise comparisons’ statistical significance was set at 2-sided 1.7% level.

## Results

### Patient characteristics

The hepatoblastoma patient group comprised 22 males (64.7%) and 12 females (35.3%), with a median age of 2 years at diagnosis (range, 0.42 to 11 years old). The BHD group comprised 18 males (60%) and 12 females (40%), with a median age of 1.84 years (range, 0.06 to 18 years old). And the NC group comprised 11 males (55.0%) and 9 females (45.0%), with a median age of 2.5 years (range, 0.42 to 18 years old). During the study period, twenty-seven of thirty-four HB patients underwent active chemotherapy. Of these, seven patients had PRETEXT stage I, two stage II, ten stage III, and eight stage IV; eleven patients had lymphovascular invasion and six lung metastasis; nine patients underwent liver transplantations; two patients had disease progression and one patient died of disease. Associated conditions included history of prematurity in nine patients, Beckwith-Wiedemann syndrome in two, Simpson Golabi Behmel syndrome in one. The BHD group included six patients with extrahepatic biliary atresia, four with Alagille syndrome, four with biliary cirrhosis, four with autoimmune hepatitis, two with infantile hemangioendothelioma, two with focal nodular hyperplasia, one with multiple dysplastic nodules, one with Wilson’s disease, one with congenital liver fibrosis, one with hepatitis C, one with type 1 tyrosinemia, and three with acute liver failure, uncertain etiology.

### GPC3 as a diagnostic marker for HB

**Figure 1:**
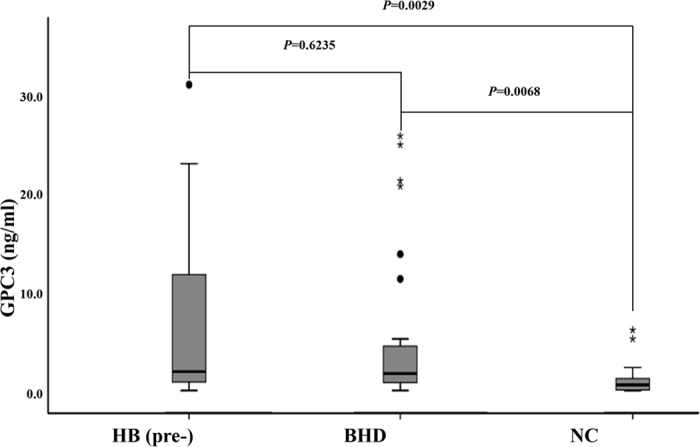
The boxplot of serum glypican 3 (GPC3) levels in pretreatment hepatoblastoma patients (HB (pre-)), patients with benign hepatobiliary disease (BHD) and normal controls (NC). The box indicates the 25th and 75th percentile values, and the line indicates the median level, whereas the interquartile range (IQR) extends outside the box. The points outside the IQR are mild outliers and the stars outside the IQR are extreme outliers. The GPC3 levels in both HB pretreatment and BHD groups were significantly higher than those in NC group (p = 0.0029 and 0.0068, respectively). However, the GPC3 levels in HB pretreatment group were not significant different from those in BHD group (p = 0.6235).

**Figure 2:**
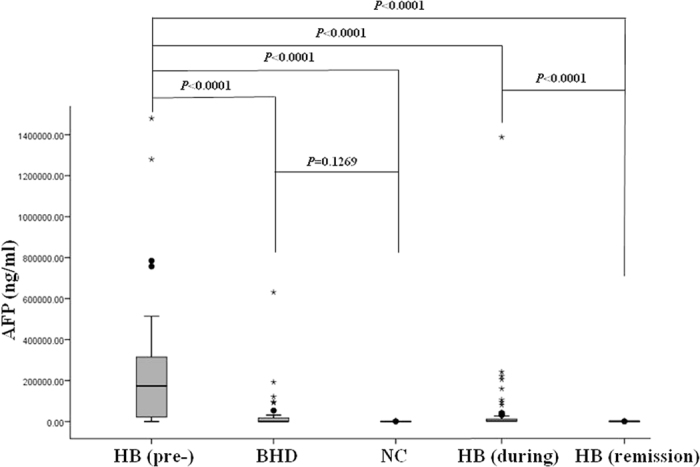
The boxplot of serum alpha-fetoprotein (AFP) levels in pretreatment hepatoblastoma patients (HB (pre-)), patients with benign hepatobiliary disease (BHD), normal controls (NC), HB patients during treatment (HB (during)) and HB patients in remission (HB (remission)). The box indicates the 25th and 75th percentile values, and the line indicates the median level, whereas the interquartile range (IQR) extends outside the box. The points outside the IQR are mild outliers and the stars outside the IQR are extreme outliers. AFP levels in HB pretreatment group were significantly higher than those in both BHD and NC groups (*P* < 0.0001 for both). And there was no significant difference in AFP levels between BHD and NC groups (p = 0.1269). There was a significant difference in AFP levels among HB pretreatment, during treatment, and remission groups (p < 0.0001 for all two comparisons).

The serum GPC3 and AFP levels in three different groups are shown in Figs 1 and 2, respectively. The median serum GPC3 levels were 1.93 ng/ml (range, 0–31.19) in HB pretreatment group, 1.74 ng/ml (range, 0–25.95) in BHD group and 0.59 ng/ml (range, 0–6.20) in NC group. There was a significant difference in GPC3 levels among these three groups (p = 0.0053 by Kruskal-wallis). As for multiple pair-wise comparisons, the GPC3 levels in both HB pretreatment and BHD groups were significantly higher than those in NC group (p = 0.0029 and 0.0068, respectively). However, the GPC3 levels in HB pretreatment group were not significantly different from those in BHD group (p = 0.6235), which indicated that GPC3 could not definitely distinguish HB from BHD. We also found some young infants in normal control group had mildly increased GPC3 levels.

The median serum AFP levels were 173,500 ng/ml (range, 64.6–1480,000) in HB pretreatment group, 38.5 ng/ml (range, 0–631,000) in BHD group, and 2.2 ng/ml (range, 0–4420) in NC group. There was a significant difference in AFP levels among these three groups (p = 0.0001 by Kruskal-wallis). Furthermore, the AFP levels in HB pretreatment group were significantly higher than those in both BHD and NC groups (*P* < 0.0001 for both). There was no significant difference in AFP levels between BHD and NC groups (p = 0.1269).

**Figure 3:**
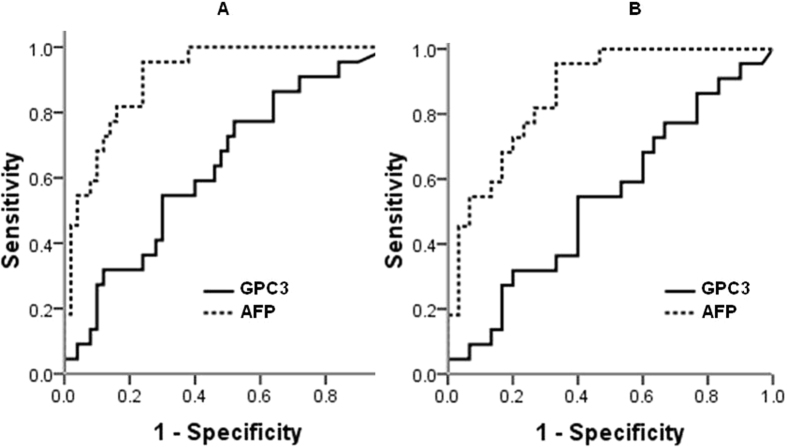
The ROC curve analysis: the diagnosis of serum glypican 3 (GPC3) and alpha-fetoprotein (AFP) for hepatoblastoma (HB) versus all controls (**A**) and HB versus benign hepatobiliary disease (BHD) (**B**). The AUROC for GPC3 and AFP was 0.63 and 0.91, respectively in A, and 0.54 and 0.87, respectively in B. AUROC indicates area under the receiver operating curve.

The areas under the receiver operating curve (AUROC) were calculated to compare the accuracies achieved when using GPC3 or AFP for diagnosis of HB. The AUROC value, sensitivity and specificity of GPC3 for pretreatment HB versus all controls were 0.63 (95% CI: 0.49 to 0.77), 54.5% and 66%, respectively, at a cutoff of 1.74 ng/mL, all significantly lower than those of AFP (0.91 (95% CI: 0.84 to 0.97), 95.5% and 76.0%, respectively) at a cutoff of 1130 ng/ml) (Fig. 3A). When it was set as pretreatment HB versus BHD only, the AUROC for GPC3 (0.54, 95% CI: 0.38 to 0.70) was also less than the AUROC for AFP (0.87, 95% CI: 0.77 to 0.96) (Fig. 3B). The findings suggest that the diagnostic value of GPC3 for HB is low and the performance of GPC3 is inferior to that of AFP.

### GPC3 as a monitoring marker for HB

**Figure 4:**
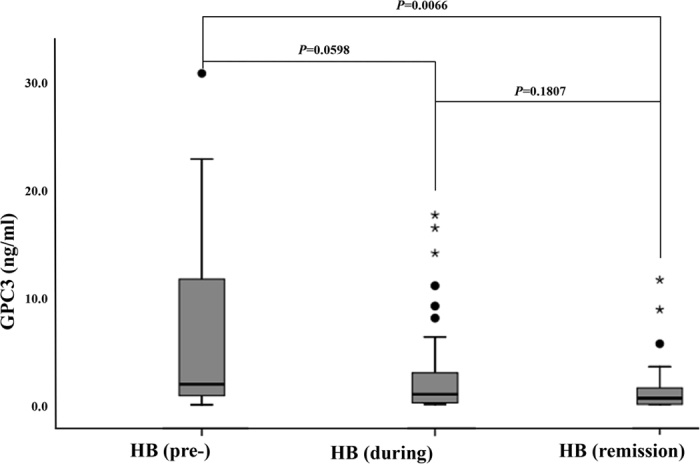
The boxplot of serum glypican-3 (GPC3) levels in pretreatment hepatoblastoma patients (HB (pre-)), during treatment (HB (during)) and in remission (HB (remission)). The box indicates the 25th and 75th percentile values, and the line indicates the median level, whereas the interquartile range (IQR) extends outside the box. The points outside the IQR are mild outliers and the stars outside the IQR are extreme outliers. There was a significant difference in GPC3 levels between HB pretreatment and remission groups (p = 0.0066). However, there were no significant differences in GPC3 levels between HB pretreatment and during treatment groups (p = 0.0598) and between during treatment and remission groups (p = 0.1807).

The serum GPC3 and AFP levels were also longitudinally monitored in HB patients. The median serum GPC3 levels were 0.94 ng/ml (range, 0–17.56) in HB during treatment group and 0.57 ng/ml (range, 0–11.54) in HB remission group. There was a significant difference in GPC3 levels among HB pre-treatment, during treatment and remission groups (p = 0.0239 by Kruskal-wallis). Besides that, there was a significant difference in GPC3 levels between HB pretreatment and remission groups (p = 0.0066). However, there were no significant differences in GPC3 levels between HB pretreatment and during treatment groups (p = 0.0598) or between during treatment and remission groups (p = 0.1807) (Fig. 4).

The median serum AFP levels were 1150 ng/ml (range, 10.9–1390,000) in HB during treatment group and 8.85 ng/ml (range, 0–817) in HB remission group. There was a significant difference in AFP levels among HB pretreatment, during treatment and remission groups (Kruskal-wallis p = 0.0001). Moreover, there were significant differences in AFP levels between pretreatment and during treatment groups (p < 0.0001); between pretreatment and remission groups (p < 0.0001); and during treatment and remission groups (p < 0.0001) (Fig. 2). The findings suggest that GPC3 is inferior to AFP as a monitoring marker for HB.

### GPC3 levels and clinicopathologic parameters

The mean and ranges of GPC3 and AFP levels in pretreatment HB patients, as based on various clinicopathologic parameters, are summarized in Table 1. Both serum GPC3 and AFP levels were not affected significantly by age, sex, tumor size, PRETEXT stage and the status of lymphovascular invasion or metastasis. In addition, we found that there was no significant correlation between serum levels of GPC3 and AFP in patients with HB (p = 0.422).

## Discussion

Glypican 3 is expressed abundantly in fetal liver, inactive in normal mature liver, and reactivated in HB and HCC. It plays an important role in cellular growth and differentiation by regulating the activities of Wnts^20^, Hedgehogs^21^, fibroblast growth factor-2^22^, and bone morphogenetic protein^23^. The *GPC3* gene was identified as one of the most overexpressed genes in HB by microarray analysis^13^. Immunohistochemical staining of GPC3 showed 100% sensitivity in HB^15^,^24^, and 83.4% sensitivity in HCC^14^. Recently, several studies evaluated serum GPC3 as a marker for HCC. Some found that GPC3 is a more sensitive and specific serum marker than AFP for HCC^16^,^17^,^18^,^25^,^26^. Conversely, others showed that GPC3 is not a good serum marker for HCC^19^,^27^,^28^,^29^,^30^. As a result, the diagnostic value of serum GPC3 in patients with HCC remains controversial. The clinical value of serum GPC3 in HB patients has not yet been investigated. In the current study, we found that the GPC3 levels in pretreatment HB group were significantly higher than those in NC group, which indicates that serum GPC3 is able to distinguish patients with HB from NCs. However, there was no statistical difference in the levels of serum GPC3 between patients with HB and patients with BHD. Moreover, the AUROCs of GPC3 for HB versus all controls and HB versus BHD were 0.63 and 0.54, respectively, suggesting that GPC3 is not an effective serum tumor marker for HB. Furthermore, the AUROC, sensitivity and specificity of GPC3 were all less than AFP. Our results suggest that GPC3 is not as good as AFP as a diagnostic marker for HB.

We also found that there were no significant differences in GPC3 levels between HB pretreatment and during treatment groups and between during treatment and remission groups, which suggest that GPC3 is not a suitable monitoring marker for HB. In contrast, there was a significant difference in AFP levels in different treatment stages of HB. Previous clinical trials have shown that age, PRETEXT stage, vascular tumor invasion, and occurrence of distant metastases are significant prognostic factors in HB patients^31^,^32^,^33^. Consistent with the observations seen in HCC patients^26^, we also found that GPC3 levels did not correlate with AFP levels, tumor size or stage. We also found serum GPC3 levels were not related to the status of lymphovascular invasion or metastasis. The findings indicate that there is little prognostic value of serum GPC3 for HB.

Previous studies have reported the discrepancy between tumor GPC3 expression and circulating levels of GPC3. Nakatsura *et al*.^18^ reported that serum GPC3 was detected in 40% (16 of 40) of patients with HCC and only in 50% (7 of 14) of patients with HCC having GPC3-positive tumor tissue. Yu *et al*.^34^ reported that serum GPC3 was elevated in 42.5% (17 of 40) of patients with HCC, and tissue GPC3 was positive in 85% (34/40) patients with HCC. In this study, all tumors expressed GPC3 with variable intensity by immunostaining. However, serum GPC3 was detected in only 13 of 22 pretreatment HB patients with a cutoff value of 1.74 ng/ml.

Several studies have confirmed the expression of GPC3 in regenerative nodules, hepatitis C samples, cirrhosis and even in normal liver tissues^19^,^35^. In a recent study, Yasuda *et al*.^27^ found the levels of serum GPC3 were higher in chronic liver disease than in HCC. Ozakan *et al*. also observed high levels of serum GPC3 in cirrhosis (median: 5.51 pg/mL) compared with HCC (median: 5.13 pg/mL)^28^. Compatible with these findings, we also observed an elevation in serum GPC3 in patients with benign hepatobiliary diseases. The underlying mechanisms are unclear. These findings suggest that GPC3 is not a specific tumor marker for either HCC or HB.

In summary, our study showed that GPC3 could not differentiate HB from BHD. The serum GPC3 had low diagnostic accuracy for HB compared with the accuracy achieved with AFP. GPC3 was not a suitable monitoring marker for HB. There was no significant correlation between GPC3 levels and the prognostic parameters assessed. We conclude that GPC3 is inferior to AFP as a serum marker for HB.

## Additional Information

**How to cite this article**: Zhou, S. *et al*. Glypican 3 as a Serum Marker for Hepatoblastoma. *Sci. Rep.* **7**, 45932; doi: 10.1038/srep45932 (2017).

**Publisher's note:** Springer Nature remains neutral with regard to jurisdictional claims in published maps and institutional affiliations.

## Figures and Tables

**Table 1:** Glypican-3 and alpha-fetoprotein levels in pretreatment hepatoblastoma patients.

	Glypican-3	p-value	Alpha-fetoprotein	p-value
	Median (rang), ng/ml		Median (rang), ng/ml	
Age, yr		0.141		0.526
<3	2.3 (0.08–31.19)		210,000 (64.6–1480,000)	
≥3	1.56 (0–13.65)		34,200 (1540–785,000)	
Sex		0.860		0.916
Male	1.82 (0–31.19)		188,000 (1540–1280,000)	
Female	2.30 (0.08–23.13)		93,700 (64.6–1480,000)	
Tumor size		0.533		0.071
<10 cm	1.3 (0.46–31.19)		71.700 (64.6–514,000)	
≥10 cm	2.3 (0–23.13)		243,000 (1540–1480,000)	
PRETEXT stage		0.503		0.062
I/II	1.56 (0.46–11.82)		22,200 (64.6–315,000)	
III/IV	2.04 (0–31.19)		210,000 (1540–1480,000)	
Lymphovascular invasion		0.291		0.644
Absence	4.34 (0.08–31.19)		199,000 (64.6–1480,000)	
Presence	1.5 (0–13.65)		87,700 (1540–1280,000)	
Metastasis		0.268		0.307
Absence	1.43 (0–31.19)		199,000 (64.6–1480,000)	
Presence	2.70 (2.04–13.65)		63,950 (1540–243,000)	
